# Effects of family-based treatment on adolescent outpatients treated for anorexia nervosa in the Eating Disorder Unit of Helsinki University Hospital

**DOI:** 10.1186/s40337-023-00879-9

**Published:** 2023-09-11

**Authors:** Svetlana Oshukova, Jaana Suokas, Mai Nordberg, Monica Ålgars

**Affiliations:** 1 Department of Psychiatry, Eating Disorder Unit, University of Helsinki and Helsinki University Hospital (HUS), P.O. Box 282, 00029 Helsinki, Finland; 2 Psychiatric Hospital, City of Helsinki, Nordenskiöldinkatu 20, 00250 Helsinki, Finland; 3 Department of Psychology and Logopedics, Faculty of Medicine, University of Helsinki, Haartmaninkatu 8, P.O. Box 63 00014, Helsinki, Finland; 4 Department of Psychology, Åbo Akademi University, Arken, Tehtaankatu 2, 20500 Turku, Finland

**Keywords:** Adolescents, Anorexia nervosa, Family-based treatment, Outpatient

## Abstract

**Background:**

Family therapy for adolescent anorexia nervosa (AN) has stronger evidence of efficacy in comparison with individual therapy, and family-based treatment (FBT) is the most evaluated in numerous randomized clinical trials. However, few studies have focused on how FBT performs outside of research settings. The current study is the first to assess clinical outcomes of FBT for adolescent AN in Finland, in a specialized outpatient clinic.

**Aim:**

The naturalistic outcome of outpatient FBT for adolescent AN was investigated.

**Methods:**

Fifty-two female patients and their families who received FBT at a tertiary eating disorders unit participated in the study. Data on their pre-treatment parameters, treatment details, and condition at the end of treatment (EOT) was collected from their medical records.

**Results:**

At EOT, a majority (61.5%) had achieved a full weight restoration [percentage of expected body weight (%EBW) ≥ 95%]. Participants with an %EBW ≥ 95 at EOT had a significantly higher pre-treatment %EBW than those with an EBW < 95% at EOT. Participants with an EBW ≥ 95% at EOT showed significantly higher total weight gain during the treatment period, a higher rate of regular menstrual periods at EOT, significantly lower rates of dietary restrictions, and less cognitive or behavioral symptoms of the eating disorder overall, compared to participants who did not achieve a normal body weight. In 22 cases (42.3%), there was no need for further treatment at the end of FBT. Participants who needed further treatment after FBT, compared to those who did not, showed significantly higher rates of psychiatric comorbidity, history of mental health treatment, and need for psychopharmacological treatment.

**Conclusions:**

In this naturalistic study, and in line with previous studies, FBT for AN appeared to be an effective and sometimes sufficient intervention, especially for patients with milder weight deficit and less severe psychiatric comorbidities. The results show that FBT can be successfully implemented in Finland and suggest that training more ED clinicians in FBT would be beneficial.

*Trial registration*: The study was retrospectively registered on February 8th, 2023, in ClinicalTrials.gov Protocol Registration and Results System, identifier: NCT05734573.

## Abstract (plain language summary)

Family-based treatment (FBT), a form of family therapy for eating disorders (ED) in young patients, has proven to be effective in numerous large-scale studies. This study aims to evaluate outcomes of the treatment for anorexia nervosa (AN) in a ‘real-world’ setting in a specialized outpatient clinic in Helsinki, Finland. Fifty-two young female patients seen for treatment between June 2013 and December 2017 were included in the study. Demographic and treatment characteristics, weight, and ED symptoms before and after treatment are reported. At the end of treatment, most patients had a good outcome, including normalization of their body weight and reduced ED symptoms. The majority of patients who needed further treatment after FBT suffered from an additional psychiatric disorder together with AN.

## Background

Eating disorders (EDs) are serious psychiatric disorders that can affect individuals of all ages and genders, but female adolescents and young adults are particularly at risk. In addition to eating behavior disturbances, the symptomatology includes mental, physical, and social impairment. EDs are associated with severe medical and psychological consequences, such as osteoporosis, growth retardation, and developmental delay in those underage [1]. Anorexia nervosa (AN) is a psychiatric disorder characterized by significantly low weight, dietary restrictions, compensatory behaviors, such as compulsive exercising and purging or laxative abuse, as well as an intense fear of weight gain and a distorted body image. Early detection and referral to treatment can reduce the length of illness and improve the prognosis remarkably [2, 3].

For adolescent AN, family therapy has stronger evidence of efficacy than individual therapy, according to systematic reviews [4, 5]; it is recommended for adolescent AN in several clinical guidelines [6, 7].

Among the family-focused treatment modes, family-based treatment (FBT) has gained the most evidence of efficacy [8]. FBT, developed in London, emphasizes parental support to the child in the process of weight restoration and normalization of eating patterns [9]. The treatment consists of three phases. Phase I, which includes an in-session family meal, focuses on weight restoration and assisting and empowering the parents in taking responsibility for their child eating. In Phase I, the therapeutic discussions focus on the management of food and eating behaviors. Phase I continues until there is steady weight restoration and the ED symptoms begin to recede. In Phase II, age-appropriate control of eating is gradually transitioned back to the adolescent. The parents are supported in managing ED symptoms until the patient can eat well on their own. The relationship between adolescent developmental issues and the ED is also explored. In Phase III, the focus lies on establishing a healthy, adolescent identity and ending treatment well. Adolescent issues are reviewed and explored, problem solving modeled, and future problems planned for [9].

FBT has been rigorously studied in randomized clinical trials (RCT) in Australia, Canada, and the United States [10–18]. The proportion of patients achieving a good clinical improvement (i.e., ≥ 85% of ideal body weight) in FBT trials varies across studies, ranging between 34 and 90% [19]. FBT response, defined as an improvement in weight and eating-related psychopathology, is reported to be at an average of about 75% (range 60–85%) [20].

While research trials have shown evidence of the efficacy of FBT, there is still a lack of studies providing evidence of the treatment’s effectiveness when delivered in routine practice settings, as well as within different national and cultural settings. Naturalistic studies reporting treatment effects in routine practice settings in different cultures and types of care would confirm the generalizability of the treatment. It would thus encourage clinicians across settings to implement the treatment. Though studies on how FBT performs in non-research settings are still few [21–25], the results of those are promising; FBT seems to be not only efficacious but also an effective treatment for adolescents with AN. A growing body of real-life study reports contributes to the evidence base for FBT as a first-line treatment method. Up to now, clinical outcomes of FBT in Finland have not been reported, and the current study aims at filling this gap.

In assessing the clinical outcomes of treatments for AN, remission is usually defined as achieving a normal body weight (≥ 95% of ideal body weight) and a reduction in eating-related symptoms, both cognitive and behavioral, to subclinical levels [15, 26]. Still, it is not easy to predict the outcome of FBT in clinical practice, as only few parameters have shown potential influence on the response to the treatment. However, early weight gain, mostly defined as a gain of 2–3 kg during the first month of treatment, has been shown to be a prognostic indicator of remission in FBT [17, 26–29].

Comorbid psychiatric disorders, the age of 18 and over, prior hospitalization, longer illness duration, as well as high parental expressed emotion (a measure of caregiver attitudes toward a relative with an illness), have been associated with lower remission rates [30, 31]. Bulimic symptoms and emotional dysregulation have been found to predict higher levels of ED symptoms post-treatment [32]. Comorbid psychiatric disorders have been shown to predict a greater chance of dropping out from FBT [31].

There are still few published reports on FBT in Northern European countries [32–34].

The primary aim of this study was to explore the effects of FBT in a naturalistic setting, in a sample of adolescents with AN referred to the Eating Disorder Unit of Helsinki University Hospital. We hypothesized that FBT would be effective in restoring body mass and reducing ED symptoms, and a sufficient intervention for some of the treated patients. We were interested in whether the results of FBT could be predicted by some pre-treatment characteristics of the patients or by the weight gain rate during the treatment. In line with previous research findings [30, 31], we hypothesized that comorbid psychiatric disorders, older age, and longer illness duration would be associated with poorer treatment outcomes. Further, we wanted to look for common features in adolescents who were still in need of psychiatric treatment after FBT.

## Methods

### Study design

The study was conducted at the outpatient department of the Eating Disorder Unit, which is a tertiary health care unit at Helsinki University Hospital’s Psychiatry Department. The unit provides specialized assessment and treatment for adolescents and adults with severe EDs in the Helsinki region, with around 1.6 million inhabitants. At the unit, treatment is primarily provided at an outpatient level, but day patient and inpatient treatments are also available. Patients and their guardians received information about the study both orally and in a cover letter. The participants were assured of the confidentiality and anonymity of the data and of the voluntary nature of participation. The patients and their guardians gave written confirmation of their consent. Data for the study was gathered from the medical records of the patients at the end of treatment.

## Participants

The participants and their parents were recruited from June 1, 2013, through December 31, 2017. All outpatients aged 13–18 years meeting the diagnostic criteria for AN and admitted to FBT at the Eating Disorder Unit of Helsinki University Hospital were invited to participate in the study. During the study period, 428 adolescent patients started outpatient treatment at the Eating Disorder unit.

Of those, 52 female patients diagnosed with AN, with a mean age of 14.50 (*SD* = 1.31) years, were admitted to FBT, and all of them participated in the study.

The pre-treatment parameters for the sample are presented in Table 1. The mean percentage of expected body weight (%EBW) at the beginning of treatment was 83.08 (*SD* = 8.58). Five patients had an EBW ≥ 95%; at pretreatment. As these adolescents still had a weight deficit in comparison to their weight before the ED onset, and presented clinically similarly to those more underweight, they were also included into the study sample. On average, the participants had suffered from ED symptoms for 13.29 (*SD* = 8.15) months before starting FBT.

**Table 1:** Pretreatment characteristics of 52 adolescent girls administered to FBT for AN

Pre-treatment parameter	N = 52
Age (years), mean(*SD*)	14.50 (1.31)
Living in an intact family, n(%)	41 (78.8%)
Living with a single parent, n(%)	5 (9.6%)
%EBW, mean(*SD*)	83.08 (8.58)
Duration of the eating disorder (months), mean(*SD*)	13.29 (8.15)
Obsessive exercise, n(%)	32 (61.5%)
Psychiatric comorbidity, n(%)	14 (26.9%)
Depression	6 (11.5%)
Anxiety disorder	3 (5.8%)
Obsessive–compulsive disorder	4 (7.7%)
Conduct disorder	1 (1.9%)
History of previous treatment for AN, n(%)	17 (32.7%)
Psychopharmacological treatment, n(%)	15 (28.8%)

## Intervention

At the Eating Disorder Unit, the aim is to offer FBT as the first-line treatment for adolescent patients with AN. However, due to the often severe symptomatology of the patients treated at the unit, as well as limited resources, only part of the patients can be admitted to FBT. On the patient’s first visit to the unit, the attending psychiatrist usually makes a clinical decision on whether FBT is suitable for the patient. The decision is based on an assessment of the patient’s medical and mental state, likelihood to commit to the treatment, and family functioning. At the time of the data collection, FBT at the unit was carried out by a family therapist experienced in the treatment of EDs, who had received certified FBT training at the Maudsley Hospital in London. Treatment fidelity was ensured by regular supervision of case material by a qualified supervisor. The duration of FBT was commonly 6–12 months, in most cases comprising 10–20 sessions. At the beginning of treatment, there were weekly sessions; later in treatment, the sessions were less frequent. The patient and their family met with their psychiatrist every four to six weeks, and the family therapist also took part in these meetings. The treatment was implemented in accordance with the FBT treatment manual [9], but in a few cases, the FBT protocol was modified to have an increased number of sessions and longer duration due to the patient’s clinical condition.

## Treatment characteristics

The number of FBT treatment sessions ranged from 5 to 40 (*M* = 15.3, *SD* = 7.34). In 11 cases (21.2%), the treatment demanded more than 20 sessions. The treatment lasted up to 23 months, with an average duration of 10.25 (*SD* = 5.0) months. In all cases but one, FBT was finished in accordance with the clinical situation and treatment plan, and only one family dropped out of treatment. This withdrawal happened after session 10 of FBT when the family decided to continue the treatment at a private clinic. As the participant had participated in half of the planned FBT sessions before dropping out and had achieved change in weight and ED symptoms, we did not exclude her from the study.

## Definition of disorders

In Finland, EDs are diagnosed in accordance with the International Classification of Diseases (ICD-10) [35]. The diagnostic criteria differ between the *Diagnostic and Statistical Manual of Mental Disorders, Fifth Edition*, (DSM-5) and ICD-10, so that the definition for EDs in DSM-5 is broader [6]. Thus, in line with previous Finnish studies [36, 37], we used a broader definition for AN. Broad AN includes ICD-10 AN (F50.0) and ICD-10 atypical AN (F50.1). An atypical AN diagnosis is used for patients in whom one or more key features of AN, such as amenorrhea or weight loss below 85% of expected, is absent, but who otherwise present a typical clinical picture.

## Assessments

Pre-treatment information about the patients’ growth and health before ED onset, as well as diagnostic evaluations of comorbid psychiatric disorders, was gathered from their medical records. Assessment at baseline and at the end of treatment (EOT) included weight, height, ED symptoms, and psychopathology. A psychiatrist performed a rigorous clinical review, which included a nonstructured interview alone with the patient, as well as with the whole family.

Validated measures were offered before FBT to all the patients for assessing ED symptoms (Eating Disorder Examination Questionnaire; EDE-Q [38]), depressive symptoms (Beck Depression Inventory; BDI [39]), and psychosocial functioning (The Clinical Impairment Assessment Questionnaire; CIA [40]).

EDE-Q, BDI, and CIA scores were only available for a small number of participants, either due to the participant not having returned them or the score not having been recorded in their medical records. Therefore, these scores were not included in this study.

According to FBT protocol, weight was assessed before every treatment session. However, only the results of the weighing at session 5 were included in the study as a marker of early response, in line with previous research [10, 28]. The participants were weighed in their underwear on a balance beam scale that was regularly re-calibrated. Height was also regularly measured.

The percentage of the expected body weight (%EBW) was calculated using national charts for age, height, and gender, according to the 50th percentile body mass index (BMI). Weight-related remission was defined as EBW ≥ 95%; partial remission was considered when EBW ≥ 85%, but < 95%; patients with EBW < 85% were considered not recovered.

The attending psychiatrists’ reports of ED symptoms and psychopathology at baseline and at EOT were used. The participants were asked about menstrual period regularity, daily meal schedule, dietary restrictions or special diets, obsessive exercise or other compensative behaviors, body-related feelings, mood symptoms, and anxiety, and the psychiatrist recorded their answers. The participants’ caregivers, as well as their therapist, were asked to express their opinion regarding whether the patient benefited from FBT or not, at the last FBT session, and this information was available from the medical records.

## Statistics

The statistical analyses were performed using IBM SPSS Statistics 25. First, the data was analyzed for descriptive parameters and incidence rates of all the variables. According to skewness and kurtosis, the data was mostly normally distributed. To assess treatment outcomes, the change in %EBW was tested using a two-way analysis of variance for repeated measures, and the McNemar chi-square test was used to analyze the change in the incidence rates of symptoms. Further, for comparing groups of patients at the EOT, Student’s *t*-test and chi-square tests were used. All analyses were two-tailed. The alpha level was set at *p* < 0.05. We also calculated Cohen’s *d* to estimate the effect sizes of statistically significant differences in the *t*-test, interpreting an effect size of 0.2 to 0.5 as small, 0.5 to 0.8 as medium, and over 0.8 as large. For the chi-square test, the phi (*φ*) coefficient was a measure for the effect size. A value of 0.1 was considered a small effect, 0.3 a medium effect, and 0.5 a large effect [41].

## Results

The findings from treatment outcomes are exhibited in Table 2. At EOT, 32 patients (61.5%) had achieved full weight restoration (> 95% EBW), 16 (30.8%) achieved partial weight-based remission (85 < %EBW < 95), and only in four (7.7%) cases EBW remained below 85%. The mean %EBW at EOT was 98.65 (10.96).

**Table 2:** Outcomes of FBT in 52 adolescent girls treated for AN

Parameter	Pretreatment	At EOT	Statistical significance of the change
%EBW, mean (*SD*)	83.08 (8.58)	98.65 (10.96)	t(df) = 13,310(51)^*^, *p* < 0.001,* d* = 1.58
Regular menses, n (%)	1 (1.9%)	28 (54.9%)	X^2^(df) = 24.083(1)^**^, *p* < 0.001,* φ* = *0.48*
Obsessive exercise, n (%)	32 (62.7%)	0 (0%)	X^2^(df) = 28.033(1)^**^, *p* < 0.001,* φ* = *0.48*
Regular meals, n (%)	5 (9.8%)	47 (90.4%)	X^2^(df) = 39.024(1)^**^, *p* < 0.001, *φ* = *0.48*
Binge eating, n (%)	3 (5.9%)	1 (1.9%)	*p* = 0.63^**^
Special diets, n (%)	17 (32.7%)	7 (13.5%)	X^2^(df) = 8.100(1)^**^, *p* < 0.001, *φ* = *0.48*

	At the EOT
85% < EBW < 95%, n (%)	16 (30.8%)
EBW ≥ 95%, n (%)	32 (61.5%)
Benefited from the FBT (parents’ assessment), n (%)	45 (88.2%)
Benefited from the FBT (therapist’s assessment), n(%)	45 (88.2%)
No dietary restrictions, n (%)	40 (76.9%)
No appearance dissatisfaction, n (%)	39 (75%)
No fear of gaining weight, n (%)	41 (78.8%)
No body image distortion, n (%)	46 (88.5%)
No need for further treatment, n (%)	22 (42.3%)

^*^ Paired samples t-test; df = degree of freedom; Cohen’s *d* used to assess the Effect Size.**McNemar Chi-Square test, X^2^ = Chi Square, df = degree of freedom, sample size N = 52; Phi (*φ)* was used to assess the Effect Size

From the parents’ point of view, as well as assessed by the therapist, 45 patients (88.2%) benefited from the treatment. At EOT, there was a statistically significant increase in the incidence of regular menstrual periods and regular meals, and a significant decrease in obsessive exercise and the proportion of participants with dietary restrictions. At EOT, 78.8% of patients reported no fear of gaining weight, 75% reported no appearance dissatisfaction, and 98.5% reported no body shape distortion.

In 30 cases (57.7%), participants continued treatment after FBT either at the Eating Disorder Unit of Helsinki University Hospital (25%, *n* = 13) or at an adolescent psychiatric outpatient clinic (30.8%, *n* = 16). A common reason for a referral to the adolescent psychiatric unit was either a comorbid psychiatric disorder or need for further, less specific psychotherapeutic support for the convalescence and age-appropriate psychological challenges. Those who continued treatment for AN at the Eating Disorder Unit, were offered a focused individual or group outpatient intervention according to their medical and psychiatric symptomatology. As mentioned above, one family chose to continue treatment at a private clinic after ten sessions of FBT.

Table 3 shows a comparison between participants who achieved a normal body weight at EOT and participants who did not. With a large effect size, the participants with a %EBW ≥ 95 at EOT had a significantly higher pre-treatment %EBW and showed a significantly higher total weight gain during the treatment period than those who did not achieve normal body weight. Compared to those who remained underweight, those achieving normal body weight at EOT showed, with large effect sizes, significantly higher rates of a regular menstrual period and significantly lower rates of dietary restrictions and cognitive or behavioral symptoms of the ED overall at EOT.

**Table 3:** Differences in pre-treatment characteristics, treatment details, post-treatment symptoms between those who achieved 95% of expected body weight at EOT and those who did not, in 52 female adolescents received FBT for AN

	%EBW ≥ 95 n = 32	%EBW < 95 n = 20	Statistical significance of the difference
Psychiatric comorbidity, n(%)	9 (28.1%)	5 (25.0%)	*p* = 1.00^*^
Time(months) before FBT, mean (*SD*)	13,38 (8.86)	13.15 (7.08)	*p* = 0.92^**^
Living in intact family, n (%)	25 (78.1%)	16 (80.0%)	*p* = 1.00^*^
Previous treatment for AN,n (%)	12 (37.5%)	5 (25.0%)	*p* = 0.38^*^
Psychiatric medication, n (%)	10 (31.3%)	5 (25.0%)	*p* = 0.76^*^
Pre-treatment %EBW,mean (*SD*)	86.9 (8.61)	76.9 (3.58)	t(df) = 4.891(50)^**^, *p* < 0.001, *d* = 1.52
Pre-treatment obsessive exercise, n (%)	23 (71.9%)	9 (45.0%)	*p* = 0.05^*^
FBT sessions number,mean (*SD*)	15.75 (7.99)	14.60 (6.28)	*p* = 0.56^**^
Early weight gain, mean (*SD*)	3.28 (2.71)	2.96 (1.74)	*p *= 0.65^**^
Total weight gain,			
mean (*SD*)	10.71 (4.77)	6.14 (1.96)	t(df) = 4.066(50)^**^, *p* < 0.001,*d* = 1.25
Regular menses at EOT, n (%)	21 (65.7%)	7 (35.0%)	X^2^(df) = 5.160(1)^***^,*p* = 0.04,φ = 0.32
Need for other treatment during FBT, n (%)	11 (34.4%)	6 (30.0%)	*p* = 1.00^*^
Need for further treatment at EOT, n (%)	17 (53.1%)	13 (65.0%)	*p* = 0.56^*^
Depression symptoms at the EOT, n (%)	9 (28.1%)	3 (15.0%)	*p* = 0.33^*^
Anxiety at EOT, n (%)	13 (40.6%)	10 (50.00%)	*p* = 0.57^*^
Dietary restrictions at the EOT, n (%)	3 (9.4%)	9 (45.0%)	X^2^(df) = 8.265(1)^***^, *p* < 0.01,φ = 0.41
No symptoms of an eating disorder at EOT, n (%)	26 (81.3%)	9 (45%)	X^2^(df) = 7.209(1)^***^, *p < *0.01,φ = 0.38

^*^ Fisher’s Exact test; ^**^ Student’s Independent Samples T-Test; t = Student’s t, df = degree of freedom; Cohen’s *d* used to assess the Effect Size; ^***^Chi Square Test; X^2^ = Chi Square, (df) = degree of freedom, Phi (*φ)* used to assess the Effect Size

Need for further treatment after FBT (Table 4) was slightly higher among participants with weight deficit at EOT (65% vs 53.1%). Participants who still needed treatment after FBT, compared to those who did not need further treatment, had significantly higher rates of psychiatric comorbidity, history of psychiatric treatment, and more need for psychopharmacological treatment. At EOT, those in need of further treatment had significantly higher rates of anxiety, depression symptoms, and ED symptoms than participants who did not need further treatment.

**Table 4:** Differences in pre-treatment characteristics, treatment details, post-treatment symptoms between those who did not need further treatment at EOT and those who needed in 52 female adolescents received FBT for AN

Parameter	No further treatment need N = 22	Further treatment need N = 30	Statistical significance of the difference
Age (years), mean (*SD*)	14.73(1.28	14.33(1.32)	*p* = 0.29
Pre-treatment %EBW, mean (SD)	80.72 (7.19)	84.81 (9.20)	*p* = 0.09
Duration of AN (months), mean (*SD*)	12.59 (6.75)	13.80 (9.11)	*p* = 0.60
Psychiatric comorbidity, n (%)	2 (9.1%)	12 (40.0%)	X^2^(df) = 6.163(1)^**^, *p* = 0.01 *φ* = *0.34*
Psychopharmacological treatment, n (%)	2 (9.1%)	13 (43.3%)	X^2^(df) = 7.250(1)^**^, *p* < 0.001*φ* = *0.37*
Previous treatment for AN, n (%)	3 (13.6%)	14 (46.7%)	X^2^(df) = 6.293(1)^**^, *p* = 0.01 *φ* = *0.35*
%EBW at EOT,mean (*SD*)	98.74 (9.87)	98.59 (11.86)	*p* = 0.96
Weight gain,mean (*SD*)	10.90 (4.53)	7.52 (3.99)	t(df) = 2.844(50)^*^, *p* < 0.001 *φ* = 0.79
Depression symptoms at EOT, n (%)	1 (4,5%)	11 (36.7%)	X^2^(df) = 7.381(1)^**^, *p* < 0.001*φ* = *0.38*
Anxiety at EOT, n (%)	1 (4.5%)	22 (73.3%)	X^2^(df) = 24.350(1)^**^, *p* < 0.001 *φ* = *0.68*
Dietary restrictions at EOT, n (%)	0 (0%)	12 (40.0%)	X^2^(df) = 10.991(1)^*^, *p* < 0.001 *φ* = *0.46*
Appearance dissatisfaction at EOT,n (%)	1 (4.5%)	12 (40.0%)	X^2^(df) = 9.902(1)^*^, *p* < 0.001*φ* = *0.45*
Fear of gaining weight at EOT, n (%)	0 (0%)	11 (36.7%)	X^2^(df) = 13.252(1)^*^, *p* < 0.001*φ* = *0.54*
Body image distortion at EOT, n (%)	0 (0%)	6 (20.0%)	X^2^(df) = 6.621(1)^**^, *p* = 0.01*φ* = *0.38*

^*^ Student’s Independent Samples T-Test; t = Student’s t, df = degree of freedom; Cohen’s *d* used to assess the Effect Size; ^**^Chi Square Test; X^2^ = Chi Square, (df) = degree of freedom, Phi (*φ)* used to assess the Effect Size

## Discussion

The aim of the present study was to evaluate the effects of FBT for adolescents with AN in terms of change in weight and ED psychopathology in a naturalistic clinical setting. Though RCT studies give clinicians guidelines for planning care and making treatment decisions, clinical reports on treatments’ naturalistic outcomes are also valuable for practitioners. To our knowledge, this is the first study on FBT for EDs in Finland, and one of very few in Northern Europe [32–34].

A majority of the participants (61.5%) achieved normal body weight (%EBW ≥ 95) at EOT. Across studies, reports on FBT response and remission rates range widely [19, 20, 27]. Response to FBT is evaluated to be about 75% (range 60–85%), defined as an improvement in weight and eating-related psychopathology. Thus, our results are in line with the research findings to date regarding weight restoration [19, 20]. In recent ‘real-life’ study reports, FBT has consistently shown its effectiveness regarding weight restoration [22–25], and our findings contribute to this evidence.

There was only one statistically significant difference in pre-treatment characteristics between those who achieved normal body weight at the end of FBT and those who did not: those who reached normal weight had a significantly higher pre-treatment %EBW than those who remained underweight. This finding is in line with previous findings showing that FBT is more effective for ED patients with less severe underweight [21, 24, 32], and that higher pre-treatment weight is a positive prognostic factor in AN patients, regardless of treatment method [21, 34, 42, 43]. Thus, in line with most studies on the subject, our findings support the importance of detecting EDs and starting treatment at an early stage to achieve a better treatment outcome. However, it should be mentioned that also low-weight and medically unstable AN patients have been shown to benefit from FBT, combined with inpatient treatment when indicated [18, 21].

In the literature, early weight gain has been shown to be a prognostic indicator of remission in FBT. We included weight gain by one month of FBT as an assessment parameter, as it has been found to be a strong early predictor of remission in previous studies [17, 26, 28]. In our study, there were no statistically significant differences in weight gain in the first weeks of treatment between those who achieved normal body weight at EOT and those who did not. One possible explanation for this might be in the differences between strict research settings and clinical practice, where the FBT protocol might be more flexible, making it more difficult to assess ‘early weight gain’ precisely [21]. Participants with a %EBW ≥ 95 at EOT showed significantly higher overall weight gain than those with a %EBW < 95 at EOT. The average weight gain in the first four weeks of FBT was approximately 3 kg in both those with a %EBW < 95 and those with a %EBW ≥ 95 at EOT, which should be prognostic of a good outcome. Thus, the difference in weight gain in treatment overall, which was statistically significant with a large effect size, was apparently due to some characteristics we did not include in the study design (except for baseline underweight severity).

We found no association between illness duration and treatment outcome. In previous research [30, 31], longer illness duration has been associated with a poorer treatment outcome. However, in recent real-life studies, results comparable to ours have been reported. Goldstein et al. [23] were not able to detect any significant predictors for weight-related treatment outcomes of FBT for adolescent AN in a private practice setting. Likewise, in a recent study on FBT effectiveness in Asian adolescents with AN [24], duration of illness, as well as the patients’ age, did not significantly predict the likelihood of weight restoration at 12 months. Further research is needed to better understand the predictors and moderators of clinical FBT outcomes.

In our study, participants with %EBW ≥ 95 at EOT showed significantly higher rates of regular menstrual periods, as well as significantly lower rates of dietary restrictions and cognitive or behavioral symptoms of the ED, at EOT. The result is not surprising. Weight gain emerged as a significant predictor of improved ED pathology and mood symptoms [44, 45]. Based on the findings of numerous studies, the British national guidelines on the recognition and treatment of EDs state that, in AN, weight gain is essential in supporting other psychological, physical, and quality-of-life changes that are required for improvement or recovery [7].

Our results strongly support the current thinking that in the treatment of AN, weight restoration should be substantial enough to achieve significant clinical improvement. Neuroscience research has demonstrated that cerebral gray and white matter deficits due to malnutrition, associated with anxiety, body image and cognitive functions, are reversible with full weight recovery in adolescents with AN [46, 47].

A clinically relevant finding of the current study was that FBT was a sufficient intervention for 42.3% of AN patients admitted to the treatment., while 57.7% required further treatment. Need for additional treatment after FBT has repeatedly been reported in previous research [10, 18]. The 22 participants who did not need further treatment after FBT showed significantly higher weight gain during FBT than those needing further treatment (*p* < 0.001), though there was no statistically significant difference in %EBW at EOT between these groups. Further, the participants who did not need further treatment after FBT had significantly lower rates of psychiatric comorbidity, less previous psychiatric treatment, and less need for psychopharmacological treatment than participants needing further treatment. At EOT, those who did not need further treatment not only showed lower rates of ED symptoms, but also lower rates of anxiety and depression, in comparison with participants who needed further treatment. In previous research, comorbid psychiatric disorders and prior mental health treatment have likewise been associated with lower remission rates [30, 31]. We found that nearly half (40%) of patients who needed further treatment after FBT had a comorbid, non-ED psychiatric diagnosis, which is in line with previous studies [48]. These participants exhibited high rates of anxiety and depression symptoms. Indeed, comorbidity demands specific clinical attention in adolescents with AN.

To our knowledge, this is the first study on FBT for AN in Finland. Several challenges in implementing FBT in Finland should be mentioned. In Finland, both parents of an adolescent are often employed full-time, which may limit their ability to provide support at mealtimes and engage in weekly FBT appointments. Young people in Finland are used to a high degree of independence in their daily activities, including meals, from a relatively young age. The idea of transferring the responsibility for daily meals from the adolescent back to parents may cause hesitance in both carers and adolescent patients. Evidence of FBT effectiveness can encourage clinicians to engage families in this type of treatment. On the other hand, there are still few trained FBT therapists in Finland, and the treatment is less familiar to clinicians than other types of ED treatments, such as individual interventions and/or intensive/day/inpatient treatment. Clinicians may feel unsure whether FBT should be the intervention of choice within their day-to-day practice, especially given the complexity of cases and limited resources. Evidence from naturalistic studies can strengthen clinicians’ confidence to implement FBT as a first-line treatment for adolescent AN. On the other hand, real-life study reports from different countries and cultures seem to reveal similarities in FBT implementation challenges, recovery rates and predictors of success [23–25].

## Strengths and limitations

The naturalistic context of the present study included both strengths and limitations. A key strength was that the sample was representative of the treatment unit. The family therapist received in-person training in FBT and supervision. A psychiatrist carefully assessed whether FBT was suitable for the participants. In planning the treatment, many factors were taken into account, including malnourishment, ED symptom severity, medical stability, psychiatric comorbidities, as well as family dynamics and parenting style.

Although FBT should be the first-line treatment for adolescent patients with AN, only 52 of 428 (12%) adolescent patients referred to the unit during the study period were admitted to FBT, which is a clear limitation of the study. A few reasons for that could be considered. As EDs are relatively common in Finland [49], Finnish healthcare presumes that before referral to a tertiary Eating Disorder unit, patients should receive assessment and assistance at the primary care level. That means that most patients referred to the present unit have severe weight deficit and serious ED symptomatology, severe comorbid non-ED psychiatric disorders, and often unsuccessful treatment attempts at a lower care level. Therefore, most patients at the unit are offered an outpatient treatment tailored for their needs, including individual or group interventions focused on weight restoration, eating behaviors, anxiety management, body dissatisfaction or other urgent issues. Family sessions, as well as nutritionist, physiotherapist or pediatrician consultations may be offered in need, and sometimes day- or full in-patient treatment is required. The often severe symptomatology of the patients treated at the unit is an important fact contributing to the relatively small percentage of patients admitted to FBT. It has been noticed in research reviews that patient clinical complexity may preclude clinicians from using FBT [19, 21].

Also, some of the patients were referred to the unit for a consultation visit and continued their treatment elsewhere. Others came when they turned 13 years old to continue treatment started at child psychiatric services where they already had received family therapy in some form, so a different type of treatment was offered to them. Some of the patients were diagnosed with bulimia nervosa and were offered other treatment approaches. Some families declined the offered FBT.

It should also be mentioned that at the time of data collection, the unit only had one family therapist, who also contributed to the unit's functioning through over types of family work. Therefore, limited resources possibly contributed to the strict selection of patients referred to FBT. The current results will guide clinical decision making at the unit, so that more patients, including patients with more severe symptoms, will be offered FBT [18].

Although the number of cases included in this study was modest, one obvious strength is the absence of dropping out from treatment. It is possible that this is related to the thorough assessment of every participant’s suitability for FBT, as well as the sufficient psychoeducation given to the families and the high professional skills of the family therapist implementing the treatment.

The data on cognitive or behavioral and mood symptoms of AN at EOT relies on a clinician’s free assessment, not on a structured interview or validated measurements. However, we consider our symptom assessments to be reliable because they were conducted by experienced clinicians who were able to detect changes in a patient’s state along the treatment process.

## Conclusions

The present study provides encouraging support for the use of FBT as an effective and well-accepted first-line treatment for adolescent AN. The majority of participants achieved normal body weight at end of treatment (EOT), and this was associated with clinical improvement. Higher pre-treatment weight was prognostic of weight restoration at EOT. FBT may be a sufficient intervention for AN patients with less severe underweight and less severe psychiatric comorbidities. Our findings support the importance of early intervention and an active focus on weight restoration in AN patients. The current results show that FBT can be successfully implemented in Finland and suggest that training more ED clinicians in FBT would be beneficial.

## Data Availability

The data are available from the corresponding author (SO) on reasonable request.

## Abbreviations

AN: Anorexia nervosa
ED: Eating disorder
BMI: Body mass index
%EBW: Percentage of the expected body weight
DSM-5: The Diagnostic and Statistical Manual of Mental Disorders, Fifth Edition
EOT: End of treatment
FBT: Family-based treatment
ICD-10: International Classification of Diseases
RCT: Randomized clinical trial
WHO: World Health Organization
